# The Phospholipid Profile of Mycoplasmas

**DOI:** 10.1155/2012/640762

**Published:** 2012-07-15

**Authors:** Jonathan D. Kornspan, Shlomo Rottem

**Affiliations:** Department of Microbiology and Molecular Genetics, The Hebrew University Hadassah Medical School, 91120 Jerusalem, Israel

## Abstract

The *de novo* synthesized polar lipids of *Mycoplasma* species are rather simple, comprising primarily of the acidic glycerophospholipids PG and CL. In addition, when grown in a medium containing serum, significant amounts of PC and SPM are incorporated into the mycoplasma cell membrane although these lipids are very uncommon in wall-covered bacteria. The exogenous lipids are either incorporated unchanged or the PC incorporated is modified by a deacylation-acylation enzymatic cycle to form disaturated PC. Although their small genome, in some *Mycoplasma* species, other genes involved in lipid biosynthesis were detected, resulting in the synthesis of a variety of glycolipis, phosphoglycolipids and ether lipids. We suggest that analyses and comparisons of mycoplasma polar lipids may serve as a novel and useful tool for classification. Nonetheless, to evaluate the importance of polar lipids in mycoplasma, further systematic and extensive studies on more *Mycoplasma* species are needed. While studies are needed to elucidate the role of lipids in the mechanisms governing the interaction of mycoplasmas with host eukaryotic cells, the finding that a terminal phosphocholine containing glycolipids of *M. fermentans* serves both as a major immune determinants and as a trigger of the inflammatory responses, and the findings that the fusogenicity of *M. fermentans* with host cells is markedly stimulated by lyso-ether lipids, are important steps toward understanding the molecular mechanisms of *M. fermentans* pathogenicity.

## 1. Introduction

Mycoplasmas (class *Mollicutes*) are the smallest and simplest self-replicating bacteria [1]. These microorganisms lack a rigid cell wall and are bound by a single membrane, the plasma membrane. Wall-less bacteria were first described 100 years ago, and now over 210 species, widely distributed among humans, animals, insects, and plants, are known. The lack of a cell wall is used to distinguish these microorganisms from ordinary bacteria and to include them in a separate class named *Mollicutes*. Phylogenetically, the *Mollicutes *are related to Gram-positive bacteria from which they developed by genome reduction [2]. Therefore, the *Mollicutes *are not at the root of the phylogenetic tree but are most probably late evolutionary products [3]. Most human and animal mycoplasmas are *Mycoplasma *species of the family* Mycoplasmataceae*. Because mycoplasmas have an extremely small genome (0.58–2.20 Mb compared with the 4.64 Mb of *Escherichia coli*), these organisms have limited metabolic options for replication and survival and depend on the host or growth medium for the supply of exogenous fatty acids, cholesterol, or complex lipids. In the present study, we shall describe and discuss the polar lipids of representative *Mycoplasma* species that belong to the *Mycoplasmataceae* family. We shall try not to overlap previous reviews on mycoplasma membrane lipids but to update information and focus on some novel and unique lipids detected in these microorganisms. Readers seeking comprehensive and comparative reviews on the mycoplasma membrane, including detailed descriptions of the composition and biosynthesis, physicochemical properties, and transbilayer distribution of mycoplasmal lipids, are referred also to the comprehensive reviews published in “Mycoplasma cell membrane” (Rottem S. and Kahane I., eds., Plenum Press, New York and London, 1993). 

## 2. Fatty Acids and Sterols Are Essential for the Cultivation of Mycoplasmas 

The dependence of mycoplasmas on an exogenous supply of fatty acids has been one of their greatest advantages as models for membrane studies [4, 5]. The ability to introduce controlled alterations in mycoplasma membrane lipids, simply by controlling the composition and content of fatty acids in the growth medium, has been used most effectively in elucidating membrane lipid organization and function in the membrane [5, 6]. In addition to long-chain fatty acids, all *Mycoplasma* species require a sterol for growth, a nutritional dependence not found elsewhere among prokaryotes [7–9]. Plant and animal sterols meet this requirement, and so do certain sterol derivatives, provided they contain the cholesterol ring system (A/B *trans*), an unsubstituted equatorial hydroxyl group, and a branched aliphatic side chain eight or more carbon atoms in length [6, 9]. For some *Mycoplasma* species, for example, *Mycoplasma capricolum,* the sterol specificity is surprisingly broad and a variety of modified cholestane derivatives satisfy, if weakly, the sterol requirement [10]. When grown in a serum supplemented growth medium, the main sterol found in mycoplasmas is unesterified cholesterol, despite the presence of excessive amounts of esterified cholesterol in medium. The low levels of esterified cholesterol incorporated from the growth medium is not required for growth and appears to form lipid droplets or pockets in the membrane [11].

The total dependence of mycoplasmas on an external supply of a sterol has been utilized to introduce controlled alterations in the sterol composition and content of the membranes, thus facilitating the analysis of the effects of sterols on membrane properties and on cell growth. The successful adaptation of the sterol-requiring *mycoides *var* capri* to grow with very little cholesterol provided a useful model system [21, 22]. The experiments carried out with the cholesterol-poor strain provided perhaps the first clear-cut evidence with membranes of growing cells to support the hypothesis promoted by Engelman and Rothman [23] that cholesterol functions as a regulator of membrane fluidity, maintaining an intermediate fluid condition during changes in growth temperature, or following alterations in the fatty acid composition of membrane lipids.

## 3. *De Novo* Synthesized Acidic Phospholipids

Gross chemical analysis of isolated mycoplasma membranes revealed that essentially all the lipids of mycoplasmas (200–400 *μ*g/mg) are located in the cell membrane. In *M. hominis* and *M. capricolum,* it was found that the lipid content depend on the growth phase of the culture, being high incells harvested at the early logarithmic phase of growth and low in stationary phase cells ([24, 25] resp.). A detailed lipid analysis of a variety of *Mycoplasma* species analyzed so far revealed that the lipid fraction contains 35–50% neutral lipids, mainly unesterified cholesterol incorporated from the growth medium, and 50–65% polar lipids [6, 26]. To demonstrate the simplicity of the lipid profile of most *Mycoplasma* species and its unique characteristics, analysis of the membrane lipids of a poorly-cultivable mycoplasma, tentatively identified as *M. hyorhinis, *was performed. This organism was first isolated from the respiratory tract of young pigs and was shown to be the major contaminant of tissue cultures [27]. Interest in *M. hyorhinis* has been recently further increased after the detection of this organism in human gastric cancer tissues, suggesting a possible association between *M. hyorhinis* and carcinogenesis [28]. When the lipid extracts from isolated membrane preparations were subjected to thin-layer chromatography (TLC) analyses, only low amounts of free fatty acids and traces of glycerides were detected. The predominant constituents of the lipid preparations were sterols and polar lipids. The main sterol found was unesterified cholesterol (80–85% of the total sterol fraction), despite the presence of excessive amounts of esterified cholesterol in the medium. The unesterified cholesterol-to-phospholipid molar ratio in *M. hyorhinis* was 1.15 similar to the ratios detected in some other mycoplasmas (Table 1).

**Figure 1:**
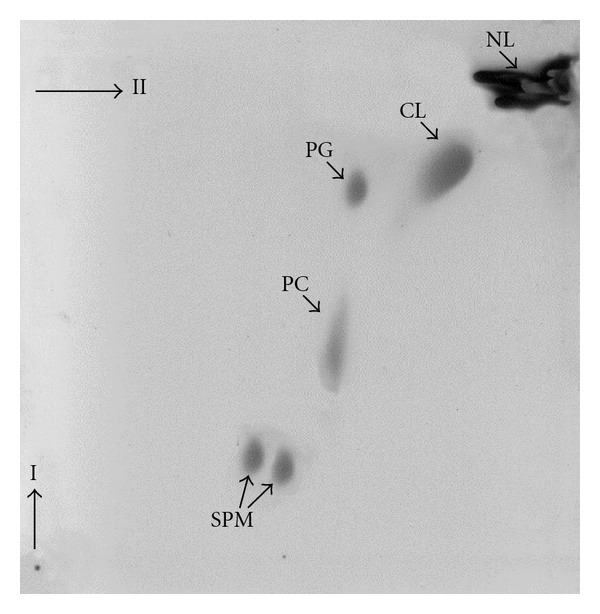
The major polar lipids of *M. hyorhinis. * Lipids were extracted from membrane preparations by the method of Bligh and Dyer [18]. The lipids were separated by TLC on silica gel plates (Kiesel-gel 60 HR, Merck, Darmstadt, Germany) developed at room temperature by a two-dimensional system using chloroform-methanol-ammonia (65 : 35 : 4 by vol.) for the first dimension and chloroform-methanol-acetic acid-water (85 : 25 : 5 : 4 by vol.) for the second dimension. Lipid spots were detected by iodine vapor, while phospholipid spots were detected by the molybdate spray reagent [29]. The spots were tentatively identified based on their comigration with commercial standards as NL, neutral lipids; CL, cardiolipin; PG, phosphatidylglycerol; PC, phosphatidylcholine; SPM, sphingomyelin.

A complete separation of the constituents of the polar lipid fraction, extracted from isolated *M. hyorhinis* membrane preparations, by 2D TLC revealed that all of the lipid spots reacted with molybdate reagent, and thus the polar lipid fraction contained exclusively phospholipids. The TLC analysis detected four major lipid spots (Figure 1). None of the lipid spots reacted with anthrone reagent, which detects glycolipids, or with ninhydrin reagent, which detects amino lipids. The compounds were tentatively identified according to their comigration on the TLC plates with commercial standards, their reaction with specific spraying reagents, and chemical analysis as sphingomyelin (SPM), phosphatidylcholine (PC), phosphatidylglycerol (PG) and cardiolipin (CL). Mycoplasmas, like other prokaryotes, are synthesizing the acidic phospholipids PG and CL. In most mycoplasmas tested so far, either PG or both PG and CL were detected [4]. In *M. penetrans* and *M. hyopneumoniae*, however, the *de novo* synthesized phospholipids consisted predominantly of CL ([12, 30], resp.). Minor *de novo* synthesized phospholipids, tentatively identified as lysophosphatidylglycerol and phosphatidic acid were also detected in several mycoplasmas [13, 24], but it seems that they represent breakdown products of PG and/or CL. In *M. gallisepticum* [14], PG, the only *de novo* synthesized acidic lipid has an unusual positional distribution of fatty acids. Fatty acids with lower melting points are located primarily at position 1 and fatty acids with higher melting points at position 2 of the *sn*-glycerol 3-phosphate. During the progression of growth from the early exponential to the stationary phase of growth, an increase in the CL-to-PG ratio in the cell membrane of *M. capricolum* was found [15, 25]. The increase in CL was almost stoichiometric with the decrease in PG and the CL accumulated upon aging was always more unsaturated than the PG. This accumulation was enhanced in palmitic acid-poor media but was inhibited even in aged cells when the cells were grown in palmitic acid-rich media, suggesting that the accumulation of CL upon aging was associated with changes in the fatty acid composition of membrane lipids [25].

 As none of the mycoplasmas tested so far are able to synthesize or modify long chain fatty acids, they depend on an exogenous supply of fatty acids in the growth medium [4]. Thus, *M. hyorhinis* could be metabolically labeled with [^3^H]-palmitate or [^3^H]-oleate (Table 2). The labeling intensity with palmitic acid was somewhat higher than the labeling intensity obtained with oleic acid. Both radioactive fatty acids were incorporated into membrane lipids with 98% of the radioactivity recovered in the polar lipid fraction representing biosynthetically labeled lipids.  Table 3 shows the relative amounts of *M. hyorhinis* polar lipids. The table also shows that PG and CL were intensively labeled by both [^3^H]-palmitate and [^3^H]-oleate whereas PC was preferentially labeled by [^3^H]-palmitate. It has previously been shown that the acidic phospholipids PG and CL are the major phospholipids synthesized* de novo* by *Mycoplasma* species [4, 6] whereas SPM is incorporated unchanged from the growth medium and PC is incorporated from the growth medium and modified by the preferential insertion of a saturated fatty acyl residue into position 2 of the *sn*-glycerol 3-phosphate, presumably by a deacylation-reacylation enzymatic sequence [14, 31].

## 4. Incorporation of Exogenous Lipids from the Growth Medium

All *Mycoplasma *species incorporate exogenous lipids mainly PC and SPM from the growth medium [26]. The SPM in all *Mycoplasma* species analyzed so far appears to be incorporated unchanged from the growth medium whereas the PC in some species is a disaturated PC, differing from the 1-saturated, 2-unsaturated PC found in the growth medium [14, 31]. In *M. gallisepticum,* it was found that the disaturated PC is synthesized by the insertion of a saturated fatty acid at position 2 of lysophosphatidylcholine (lyso-PC), derived from exogenous PC of the growth medium, by what appears to be a deacylation-acylation enzymatic sequence [14]. The modification of the exogenous PC by *M. gallisepticum* was inhibited by chloramphenicol under conditions that did not affect *de novo* synthesis of PG. The PC modification of *M. gallisepicum *was also affected by the fatty acid composition of the exogenous PC species. Di-unsaturated, 1-saturated-2-unsaturated, and 1-unsaturated-2-saturated PCs were modified to various extents, whereas the disaturated dipalmitoyl PC (DPPC) was not. Both modified and unmodified PCs were incorporated by the cells, but the unmodified DPPC was incorporated at a lower rate and to a lesser extent [31].

The ratio of SPM to PC in *M. hyorhinis* was ~2.6, much higher than the ratio found in the growth medium (0.3–0.6). Comparing this ratio to the SPM to PC ratios reported in other *Mycoplasma* species whose lipids were thoroughly analyzed and genomes completely sequenced (Table 3) revealed that whereas in *M. hyorhinis* and *M. penetrans *the SPM to PC ratio was high, in *M. gallisepticum *and *M. fermentans* the ratio was similar to the ratio found in the growth medium [12–14]. Interestingly, the genomes of *M. hyorhinis* and *M. penetrans* (GenBank: CP002669.1 and NC_004432.1 resp.) unlike the genomes of *M. gallisepticum* and *M. fermentans* (GenBank: AE015450.2 and CP001995.1 resp.) encode a CL synthetase (GenBank: AEC45753.1 and NP_757669.1 resp.) and accordingly possess a high CL to PG ratio in their polar lipid fraction (3.35 and 2.29, resp.). It is tempting to assume that since CL in the presence of divalent cations tends to form inverted hexagonal phase structures and the balance between lipids forming lamellar and hexagonal structures must be kept within certain limits, the increased capacity of CL containing *Mycoplasma* species to incorporate exogenous lipids, mainly SPM, is a consequence of a regulatory attempt aiming to preserve the bilayer stability, maintaining the properties of a permeability barrier. It is interesting to note that in *M. mobile,* though its genome encodes a CL synthetase (GenBank: AAT27942.1), the SPM to PC ratio was low (0.64; [38]). Nonetheless, the activity of the CL synthetase of this organism seems to be low, as indicated by the low CL to PG ratio in* M. mobile *membrane lipids [38]. 

The importance of SPM as a growth factor for the cultivation of a mycoplasma from the *Spiroplasmataceae* family (*Spiroplasma citri*) was previously established [41], supporting our notion that the incorporation of SPM by *M. hyorhinis* is critically important for the proper packing of membrane lipids. 

In *M. capricolum,* the incorporation of the exogenous phospholipids had essentially no effect on the rate of cell growth and did not decrease the overall phospholipid biosynthesis of the cells. Thus, the ratio of phospholipid to protein in membranes from cells grown with 5% horse serum was 0.5 (*μ*mol/mg) compared to 0.3 (*μ*mol/mg) in cells grown without serum, and the relative content of charged polar lipids was apparently decreased. The consequence of the incorporation of exogenous PC was an alteration in the relative amount of the major end-products of the *de novo* phospholipid biosynthesis; a marked increase in the ratio of CL to PG was observed. The physiological function of the PG-to-CL conversion is not known. However, since CL may be induced in the presence of cytosolic Ca^+2^ to form nonlamellar phases [42, 43], it has been suggested that the CL-to-PG increase is part of a control mechanism to maintain an intermediate membrane lipid structure [15, 44]. These structures would contain a balanced mixture of bilayer and nonbilayer lipids that would have to satisfy the structural role as well as participate in various membrane-mediated processes [44, 45]. 

## 5. Unique Polar Lipids in *Mycoplasma* Species

In the human pathogen *M. pneumoniae*, although its very small genome [46], the organism has a substantial capacity for glycolipid biosynthesis [47], yielding three glycolipids and five phosphoglycolipids. The structure of the major glycolipid, *β*-1, 6Glc-*β*-Gal-DAG, was established by NMR spectroscopy, and it was shown that this glycolipid is an important antigen in early infections [47]. 

**Figure 2:**
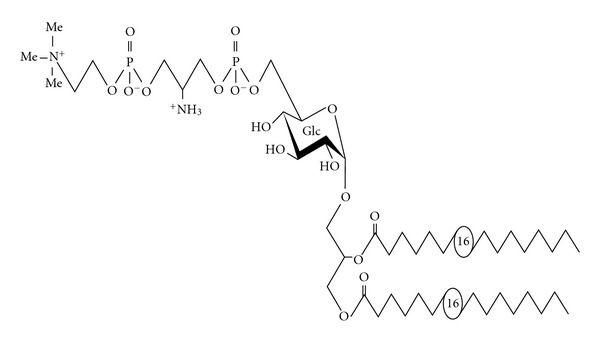
Structure of MfGL-II of *M. fermentans*. The chemical structure of the major polar lipid of *M. fermentans*, was investigated by GLC-MS, MALDI-TOF mass spectrometry, as well as one-and two-dimensional homo- and heteronuclear NMR spectroscopy and identified as 6′-*O*-[3*″*-phosphocholine-2*″*-amino-1*″*-phospho-1*″*,3*″*-propanediol]-*α*-D-glucopyranosyl-(1′→3)-1,2-diacyl-glycerol (MfGL-II). Palmitate (16 : 0) and stearate (18 : 0), in a 3.6 : 1 molar ratio, constitute the major fatty acids present [32].

Unique polar lipids were also detected in *M. fermentans* [37, 48]. This organism was first isolated from the human urogenital tract and since then its role as pathogen and cofactor in diverse diseases has considerably emerged, such as its role in the pathogenesis of rheumatoid arthritis [49]. Although little is known of the molecular mechanisms underlying *M. fermentans* pathogenicity, there is increasing evidence that the interactions with host cells are mediated by components of the plasma membrane [49, 50]. Matsuda et al. [51, 52] characterized two glycolipids (GGPL-I and GGPL-III) from *M. fermentans* strain PG18. GGPL-I structure was identified as 6′-*O*-phosphocholine–D-glucopyranosyl-1,2-diacyl-*sn*-glycerol [52]. Both GGPL-I and GGPL-III share the basic structure but differ in their polar head groups [52]. The GGPLs, synthesized from diacyl glycerol [53], were shown to be species-specific major lipid antigens of *M. fermentans* [54]. Later on, a complete structural analysis of a phosphocholine containing glycolipid (MfGL-II) isolated from *M. fermentans* strain JER was presented [32, 48]. This phosphoglycolipid is the major *de novo* synthesized polar lipid in the membrane of *M. fermentans* strain JER and accounts for 60–70% of membrane lipid phosphorous [48]. The structure of MfGL-II was elucidated by mass and NMR spectroscopy and identified as 6′-*O*-[(3*″*-phosphocholine-2*″*-amino-1*″*,3*″*-propanediol)-*α*-D-glucopyranosyl]-(1′→3)-1,2-di-palmitoyl-*sn*-glycerol (Figure 2; [32]). MfGL-II shows high structural homology to GGPL-I [52], and, in both MfGL-II and GGPL-I the phosphocholine moiety is the terminal structural motif. The main difference between GGPL-I and MfGL-II is the presence of a 2-amino-1,3-propanediol moiety and an additional phosphate residue. These data and those presented by Matsuda et al. [52] suggest that GGPL-III and the MfGL-II could be structurally identical compounds.

A gene (MFE_01510) encoding the cholinephosphotransferase in the biosynthesis of the phosphocholine-containing glycolipids was identified in our genomic analysis [34] as well as in the genome of *M. fermentans* PG18 [55]. The gene comprises an open reading frame of 762 bp encoding 254 amino acids. It has a 27% amino acid identity with LicD of *Haemophilus influenzae *(GenBank: P14184) and 26% identity with LicD of *Streptococcus pneumoniae* (GenBank: CAI34638). Out of the 23 *Mollicute* genomes sequenced so far and deposited in the GenBank database, CDSs homologous to LicD were detected in *M. pulmonis* (GenBank: NP_325836) and *M. arthritidis* (GenBank: ACF07060). The physicochemical characteristics of MfGL-II suggested that this molecule is an amphiphilicmolecule with a nonlamellar cubic aggregate structure corresponding to a conical conformation of the single molecules [56].

**Figure 3:**
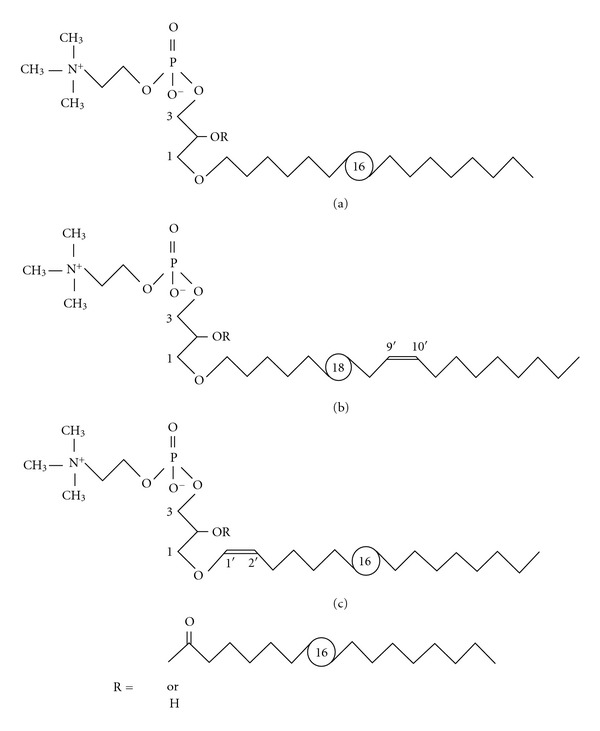
Structures of ether lipids of *M. fermentans*. Both MfEL (R=acyl) and lyso-MfEL (R=H) are mixtures of different molecular species with respect to the alkyl/alkenyl residues which are hexadecyl (a), (*9Z*)-octadec-9′-enyl (b), or (*1Z*)-alk-1′-enyl (c) [37].

The classical acidic phospholipids PG and CL account for only 15–20% of the total lipid phosphorous of *M. fermentans* but in addition to the phosphocholine containing glycolipids low amounts (~5%) of two ether lipids (Figure 3), 1-*O*-alkyl/alkenyl-2-*O*-acyl-glycero-3-phosphocholine (MfEL) and their lysoform 1-*O*-alkyl/alkenyl-glycero-3-phosphocholine (lyso-MfEL; [37]) were described. The ether lipids are heterogeneous with respect to both acyl and alkyl/alkenyl residues. The acyl residues at position 2 of glycerol are hexadecanoyl and octadecanoyl in a molar ratio of 3.6 : 1 with a trace amount of octadecenoyl. The alkyl/alkenyl residues at position 1 of glycerol are hexadecyl (78%), octadecyl (7%), octadecenyl (14%), and hexadecenyl (a trace amount). In the predominant alkenyl (octadecenyl) residue, the double bond has the *cis* configuration and is located at position 1′ (plasmalogen-type lipid) or 9′ in a ratio 1 : 1. Lipids of this type have been found in some Gram-positive bacteria, thus supporting the concept of their close taxonomical relation to mycoplasmas [37]. 

## 6. Biological Activities of the Phosphoglycolipids and Ether Lipids 

As the phosphocholine containing glycolipids constitute the major lipid fraction of *M. fermentans* membrane, it appears likely that phosphocholine is a key structure in cellular adhesion of *Mycoplasma* to host cells. Indeed, anti-MfGL-II antisera inhibit the attachment of *M. fermentans* to host eukaryotic cells suggesting that MfGL-II plays a major role in *M. fermentans-*host cell interaction [50]. These findings were supported by Matsuda and coworkers who showed that the phosphocholine containing glycolipids are major immunodeterminants of *M. fermentans* [54, 55]. Furthermore, it was also observed that MfGL-II governs the stability and permeability properties of *M. fermentans *[56] and triggers inflammatory responses, like activation of protein kinase C and the secretion of nitric oxide and prostaglandin E_2_ [40]. MfGL-II was found to induce cytokines such as tumor necrosis factor-*α* (TNF-*α*) in human mononuclear cells, although to a significantly lower degree than LPS [56]. 

**Figure 4:**
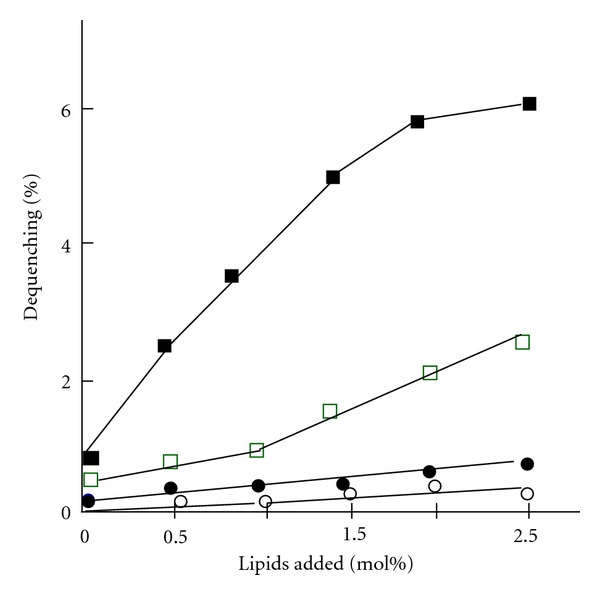
The effect of *M. fermentans* lipids on the fusion of SUV with Molt-3 cells. Small unilamellar vesicles (SUVs) were prepared by sonicating a buffer solution containing egg-phosphatidylcholine (about 5 mg per mL) with various amounts of the glycophospholipid MfGL-II or the ether lipids MfEL or lyso-MfEL [37] as previously described [39]. The SUVs were fluorescently labeled with the octadecyl rhodamine B chloride (R18). Fusion of SUV with T-lymphocytes of the human CD4^+^ Molt-3 was monitored by following the fluorescence intensity of the Molt-3 cells, and the results were presented as % dequenching [40]. (○): SUV alone; (●): SUV + MFGL-II; (□): SUV + MfEL; (■): SUV + lyso-MfEL.

The lack of a rigid cell wall allows direct and intimate contact of *M. fermentans* membrane with the cytoplasmic membrane of the host cell. Under appropriate conditions, such contact may lead to cell fusion [49, 57]. Fusogenicity was stimulated by Ca^+2^ ions and depends on the proton gradient across the mycoplasma cell membrane, decreasing markedly when the proton gradient is collapsed by proton ionophores [39, 49]. During the fusion process, mycoplasmal components are delivered into the host cell and affect the normal functions of the cell. In this context, the constituents of the plasma membrane of *M. fermentans* were intensively studied in order to provide greater insights into the molecular basis of the fusogenicity. Whereas the phosphoglycolipid MfGL-II has no fusogenic properties [49], the ether lipids, mainly lyso-MfEL, have a marked effect on the fusion of liposomes with host eukaryotic cells (Figure 4).

## Figures and Tables

**Table 1:** Phospholipids and cholesterol in the cell membrane of representative *Mycoplasma* species.

*Mycoplasma* species	PL	C	CE	C/PL (molar ratio)	Reference
	(nmole/mg membrane protein)		
*M. hyorhinis*	205.0	235.3	51.7	1.15	—
*M. penetrans*	125.5	157.1	22.3	1.25	[12]
*M. fermentans*	203.5	150.0	50.0	0.99	[13]
*M. gallisepticum*	167.0	76.0	4.7	0.48	[14]
*M. capricolum*	227.0	75.0	72.3	0.64	[15]

*M. hyorhinis* was grown in a modified Hayflick's medium [16] to the mid-exponential phase of growth, and isolated membranes were prepared as previously described [17]. Lipids were extracted from membrane preparations by the method of Bligh and Dyer [18]. Unesterified cholesterol, cholesterol esters, and phospholipids were separated on Merck Silica Gel G glass plates using benzene : diethyl ether : ethanol : acetic acid (50 : 40 : 2 : 0.2). The unesterified cholesterol and the cholesterol esters spots were extracted with chloroform for 30 min at room temperature, and the sterol content was determined by the phthaldialdehyde method [19]. Phosphorus in the phospholipid spot was determined by the method of Zhou and Arthur [20] using KH_2_PO_4_ as a standard. In brief, the spots were scraped from the plate into test tubes, digested with 0.5 mL of 70% perchloric acid (HClO_4_), and transferred into a 2 mL solution containing malachite green (0.2%) and ammonium molybdate (4%) in Tween-20 (1.5% w/v) and 5 M HCl (3 : 1 by vol.). The results are the average of three independent experiments using different batches of cells. PL: total phospholipids; TC: total cholesterol; C: unesterified cholesterol; CE: cholesterol esters.

**Table 2:** The incorporation of radiolabelled fatty acids into the major phospholipids of *M. hyorhinis. *

Tentative identification	Lipid phosphorus		Radioactivity (% of total)	
	*μ*g/mg protein	% of total	[^3^H]-palmitate	[^3^H]-oleate
SPM	6.6 ± 1.5	33.5 ± 6.7	1.0 ± 0.5	0.0 ± 0.0
PC	2.4 ± 0.5	12.6 ± 2.0	40.0 ± 3.5	19.0 ± 2.8
PG	2.4 ± 0.6	12.4 ± 3.1	12.4 ± 1.1	20.7 ± 5.8
CL	8.4 ± 1.3	41.5 ± 6.4	46.6 ± 4.4	60.0 ± 8.8

*M. hyorhinis* cells were grown in the presence of either [^3^H]-palmitate or [^3^H]-oleate. Lipids were extracted by the method of Bligh and Dyer [18] and separated by TLC on silica gel plates (Kiesel-gel 60 HR, Merck, Darmstadt, Germany) developed at room temperature by a two-dimensional system described above. The lipid spots were scraped off the plates and analyzed for radioactivity. To determine phosphorus in the phospholipid spots, the method of Zhou and Arthur [20] was used. The results are the average of three independent experiments using different batches of cells. SPM: sphingomyelin; PC: phosphatidylcholine; PG: phosphatidylglycerol; CL: cardiolipin.

**Table 3:** *De novo* synthesized phospholipids and phospholipids incorporated from the growth medium in representative *Mycoplasma* species.

Organisms	Lipid phosphorus (% of total)		Lipid phosphorus ratio		CLS	Reference
	SPM	PC	SPM/PC	CL/PG
*M. gallisepticum*	16.5	25.5	0.64	−	−	[14, 33]
*M. fermentans*	3.9	6.4	0.61	−	−	[13, 34]
*M. hyorhinis*	33.5	12.6	2.66	3.35	+	[35]
*M. penetrans*	50.0	6.2	8.06	2.29	+	[12, 36]

*M. hyorhinis* lipids were extracted, separated by TLC, and analyzed for phosphorus as described in Table 2. SPM: sphingomyelin; PC: phosphatidylcholine; PG: phosphatidylglycerol; CL: cardiolipin; CLS: cardiolipin synthetase.
